# Supplementary material for: Computational inference of cancer-specific vulnerabilities in clinical samples
Source: Genome Biol. 2020 Jul 27;21:155. doi: 10.1186/s13059-020-02077-1 (PMC7386251; doi:10.1186/s13059-020-02077-1)
Supplement: Supplementary file 1 — Additional file 1: Figure S1. Prediction performance by precision and recall. Figure S2. Prediction performance with the ARACNe network. Figure S3. Comparison of simulation results with experimental perturbation data in MCF7. Figure S4. Differential dependency of tumor versus normal samples on genes with copy number changes. Figure S5. Differential dependency of tumor versus normal samples on genes with point mutations. Figure S6. Differential expression of some alternative NHEJ genes in tumor versus normal samples. Figure S7. Additional Annexin V and BrdU data for RAN. Figure S8. Additional Annexin V and BrdU data for XRCC6. Figure S9. Additional Annexin V and BrdU data for PSMB4. Figure S10. Images of the full uncropped scans for Fig. 7a and Fig. 7b. Figure S11. Increases in the level of proapoptotic proteins by PSMB4 knockout. Figure S12. Changes in DNA-PKcs expression in response to XRCC6 knockdown. [file 13059_2020_2077_MOESM1_ESM.pdf]

Figure S1

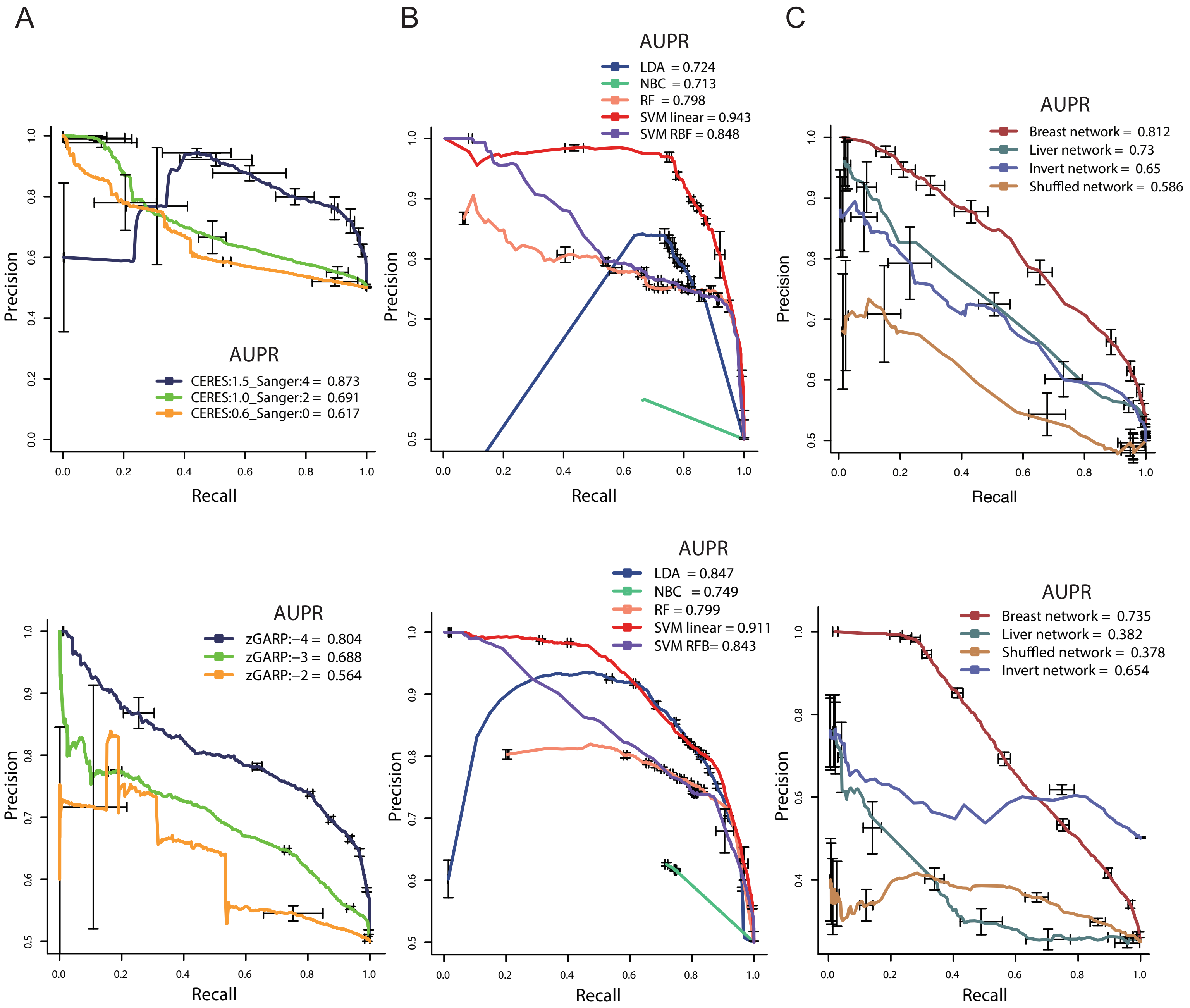

Figure S1. Prediction performance by precision and recall.
(A) Precision-recall curves from our DNN model according to different cutoffs of the cell line screen data. (B) Precision-recall curves derived from different prediction models when the most stringent cutoff was used for training. (C) Precision-recall curves from the DNN model with different test networks. We chose genes that have > 100 downstream genes in the regulatory network and used their perturbed expression patterns as training input. As negative controls, the breast cancer network was randomized by shuffling the nodes while maintaining the network structure (shuffled network) or inverted by reversing the orientation of all links (inverted network). In addition, the same types of networks were constructed by using liver cancer data (liver network). Shown are the averages of the five best models.

Figure S2

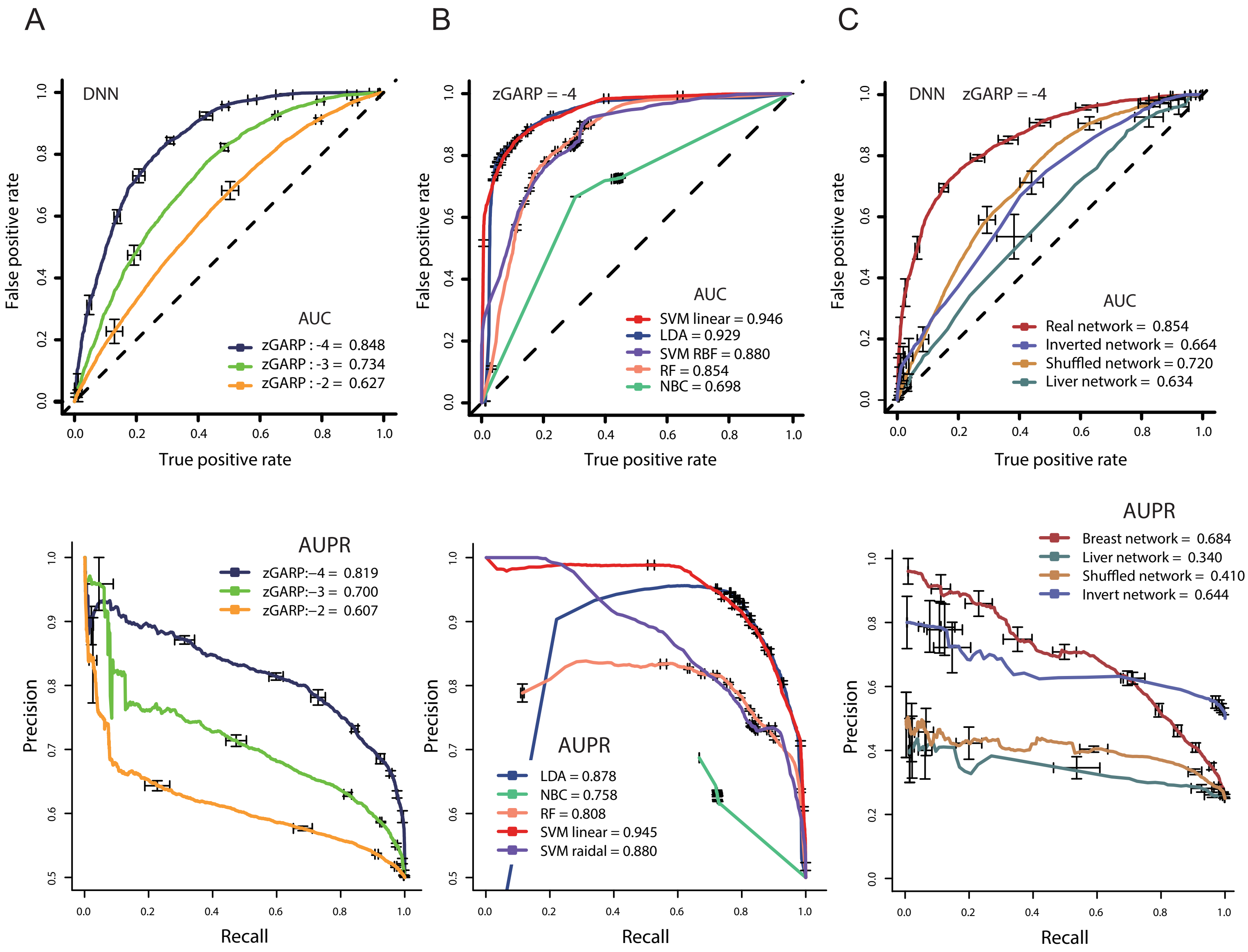

Figure S2. Prediction performance with the ARACNe network.
(A) ROC curves (upper) and precision-recall curves (lower) from our DNN model based on the ARACNe network according to different cutoffs of the RNAi screen data. (B) ROC curves (upper) and precision-recall curves (lower) from different prediction models when the most stringent cutoff was used for training. (C) ROC curves (upper) and precision-recall curves (lower) from the DNN model with different test networks. We chose genes that have > 100 neighboring genes in the ARACNe network and used their perturbed expression patterns as training input. As negative controls, the breast cancer network was randomized by shuffling the nodes while maintaining the network structure (shuffled network) or inverted by reversing the orientation of all links (inverted network). In addition, the same types of networks were constructed by using liver cancer data (liver network). Shown are the averages of the five best models.

Figure S3

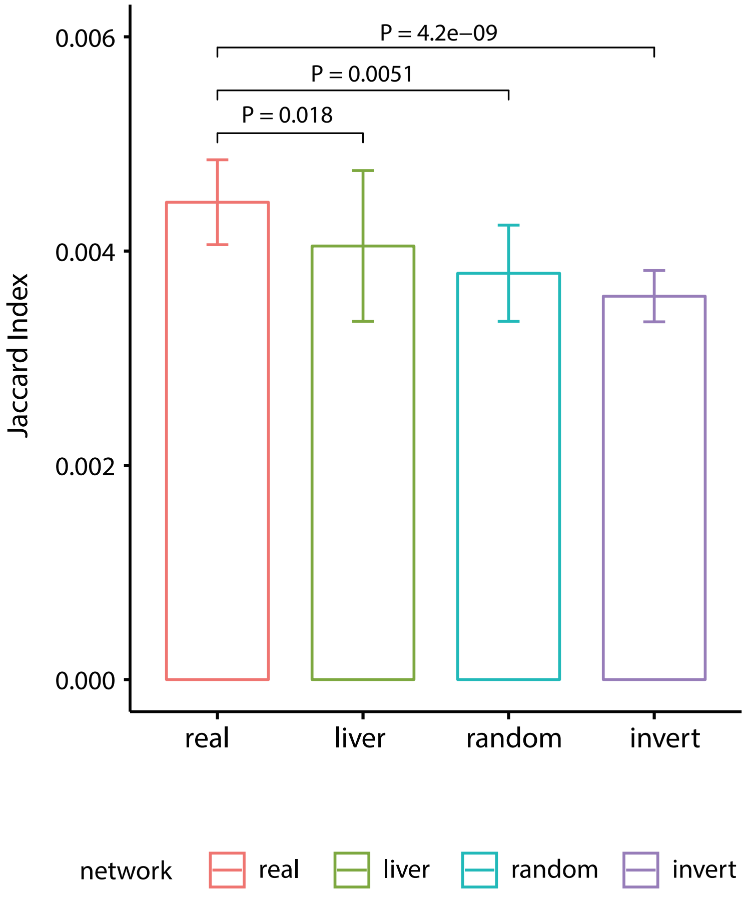

Figure S3. Comparison of simulation results with experimental perturbation data in MCF7.
Since it is difficult to estimate the fraction of expression changes that can be captured experimentally, we sought to compare the four regulatory networks instead of absolutely assessing the overlaps between the simulated and experimental results. We used the Jaccard index as a measure of the overlap that two sample sets share (defined as the size of the interaction divided by the size of the union of the sample sets). We identified genes that were up- or down-regulated by knockdown of each RNAi target gene. The up- and down-regulated genes were matched separately. The Jaccard correlation was obtained between simulation results derived from the four different networks and experimental results in MCF7 from the L1000 Connectivity Map perturbation profiles (GSE92742)..

Figure S4

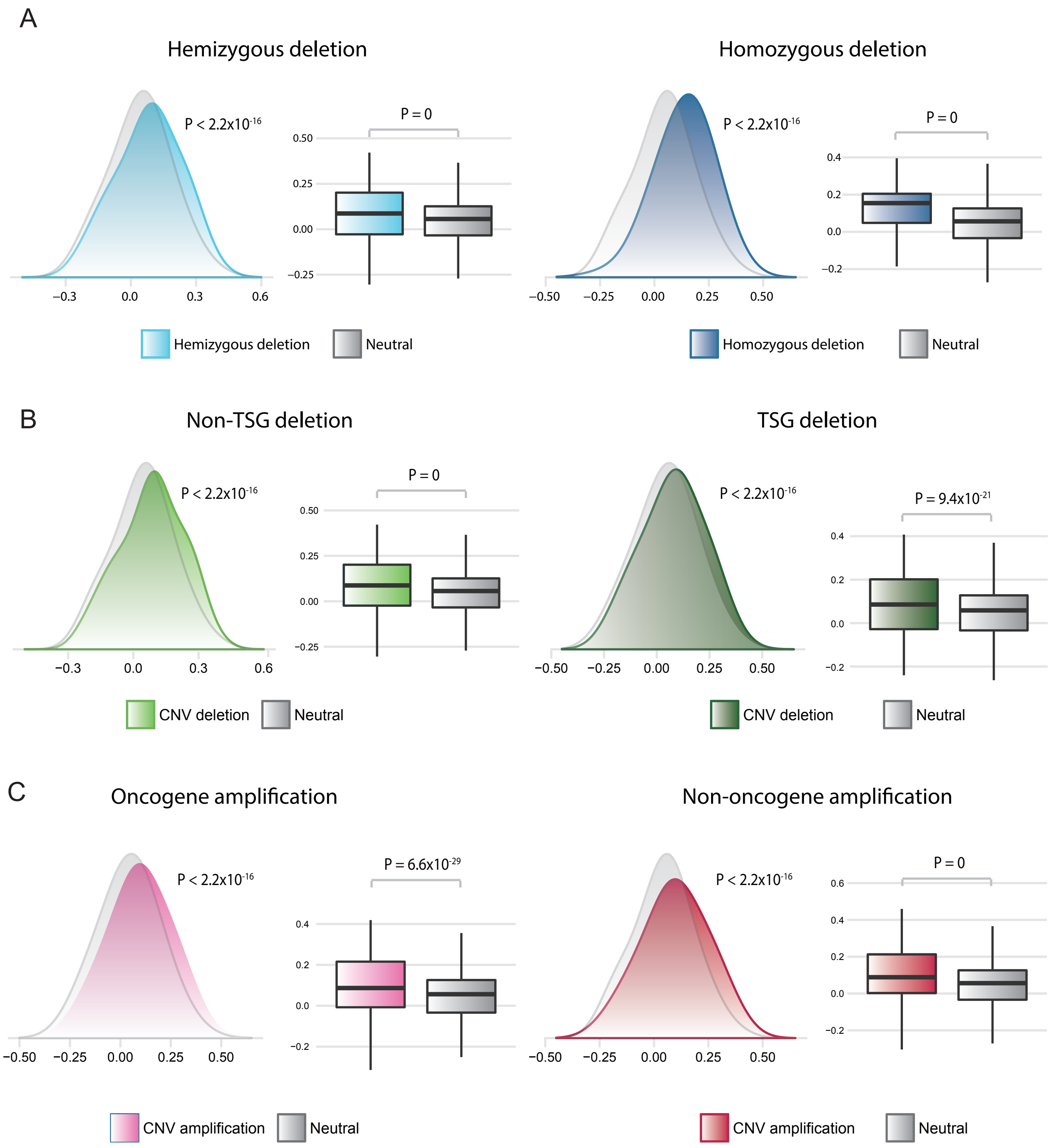

Figure S4. Differential dependency of tumor versus normal samples on genes with copy number changes. (A) Differential dependency on genes with hemizygous deletion (left) or homozygous deletion (right) in mutant (colored) versus wild-type (grey) samples. (B) Differential dependency on non-TSGs (left) or TSGs (right) with copy number deletion in mutant (colored) versus wild-type (grey) samples. (C) Differential dependency on known oncogenes (left) or non-oncogenes (right) in mutant (colored) versus wild-type (grey) samples. Differential dependency was defined as the degree of an increase in DNN prediction scores in tumor samples relative to matched normal samples. P values attached to the density plots are based on the Kolmogorov–Smirnov test. P values over the box plots are from the Student's t-test.

Figure S5

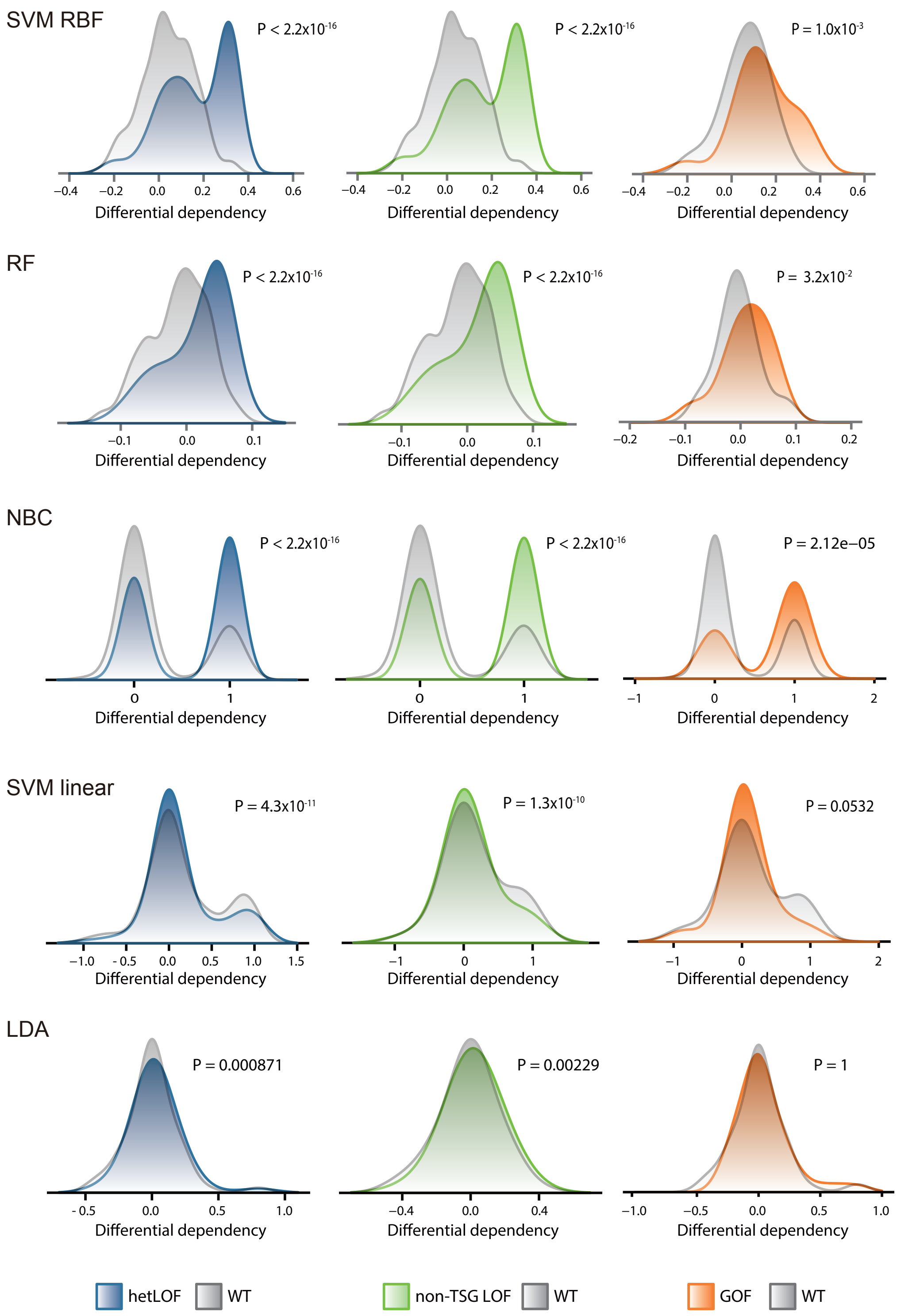

Figure S5. Differential dependency of tumor versus normal samples on genes with point mutations. Differential dependency on genes that carry hetLOF mutations (left), non-TSGs that carry LOF mutations (middle), and genes that carry GOF mutations (right) in mutant (colored) versus wild-type (grey) samples as estimated by radial SVM, RF, NBC, linear SVM, and LDA. Differential dependency was defined as the degree of an increase in the prediction scores in tumor samples relative to matched normal samples. P values are from the Kolmogorov–Smirnov test.

Figure S6

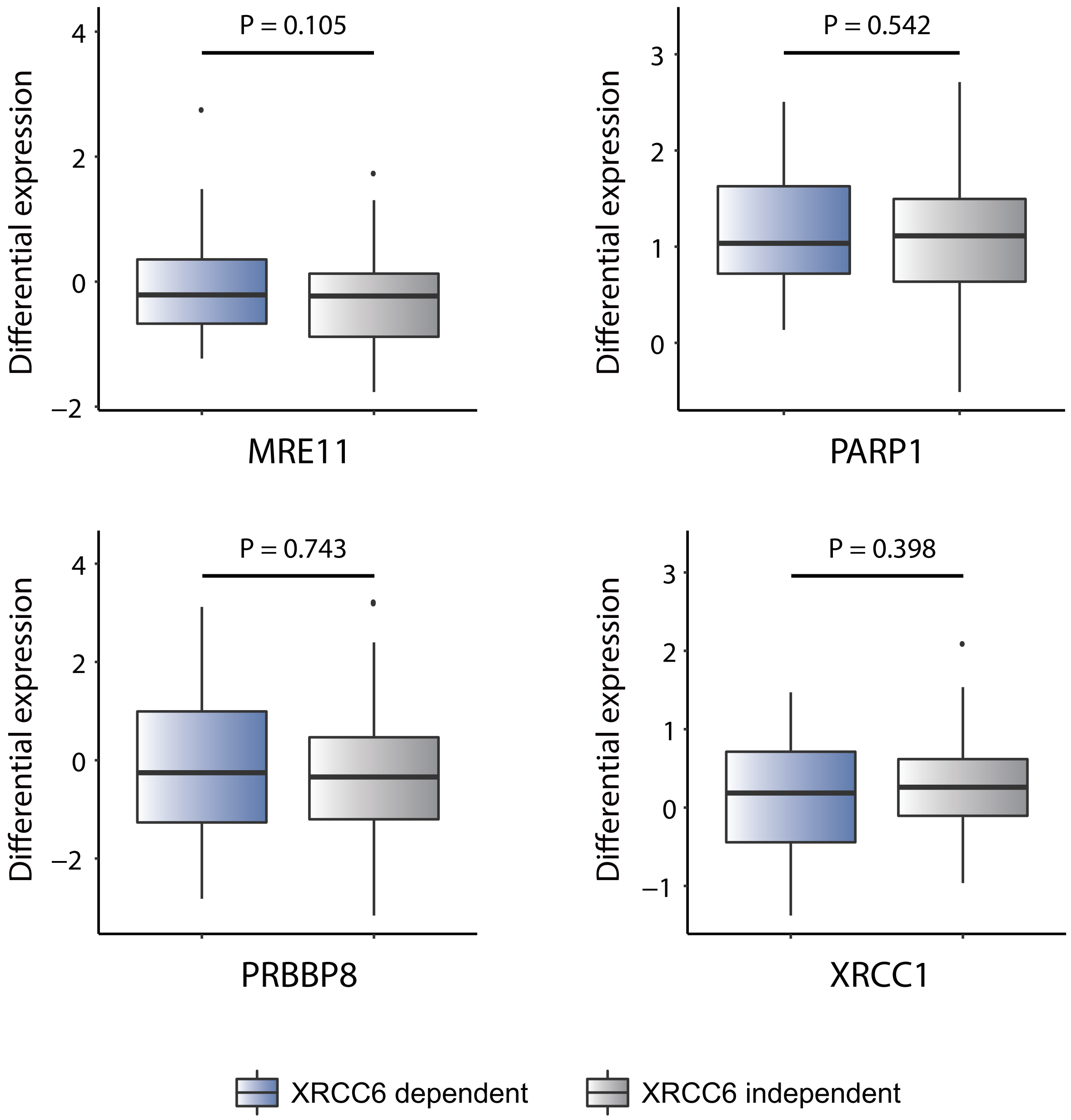

Figure S6. Differential expression of some alternative NHEJ genes in tumor versus normal samples. DNN-estimated dependency on XRCC6 is marked in blue. Differential expression of a few alternative NHEJ genes in tumor relative to normal samples is compared between the XRCC6-dependent versus independent samples.

Figure S7

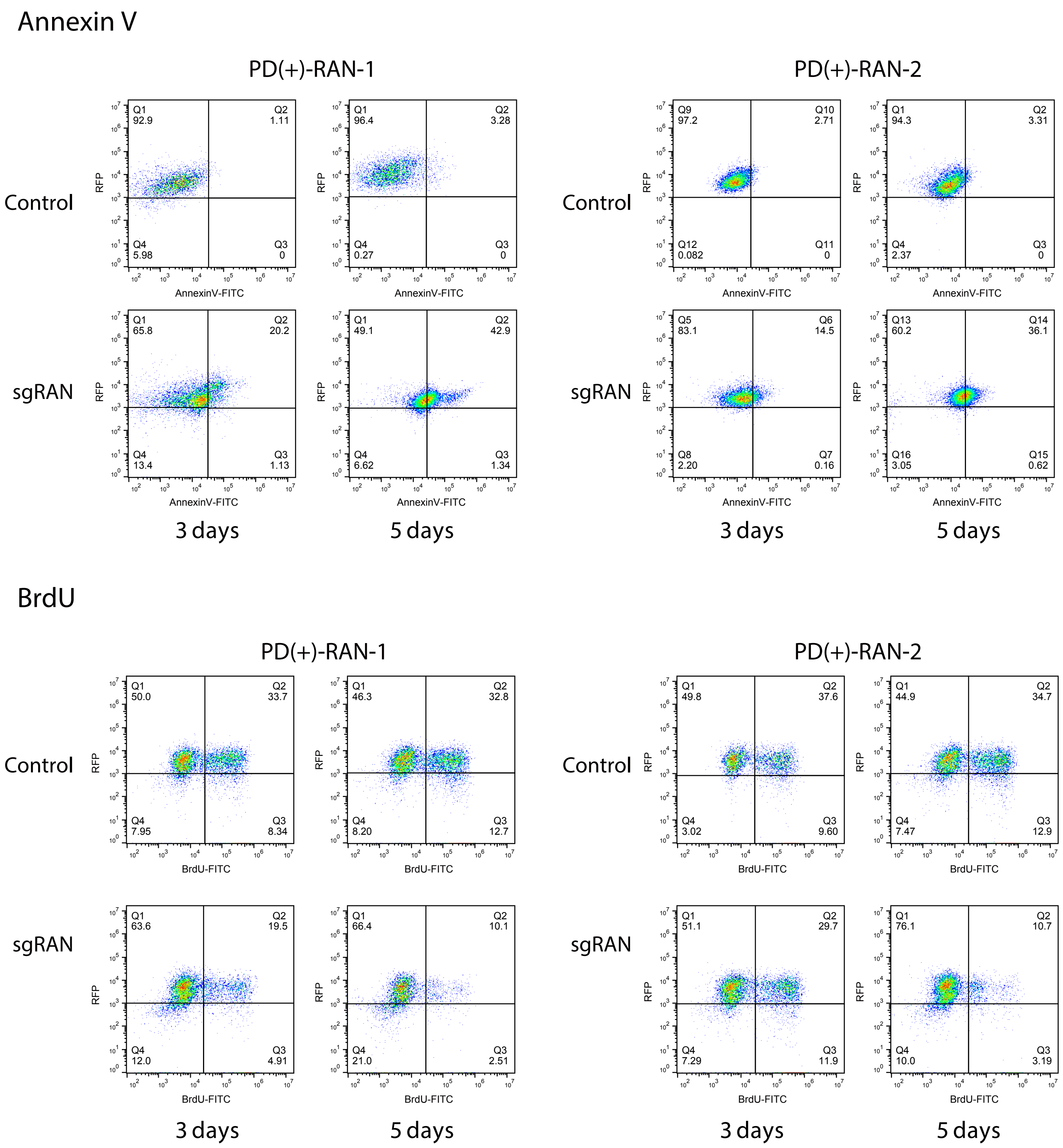

Figure S7. Additional Annexin V and BrdU data for RAN.
Scatter plots for Annexin V and BrdU staining in PD(+)RAN-1 and PD(+)RAN-2 before and after RAN inactivation. Shown here are results on day 3 and 5 after inactivation. Two sgRNAs were used simultaneously.

Figure S8A

Annexin V

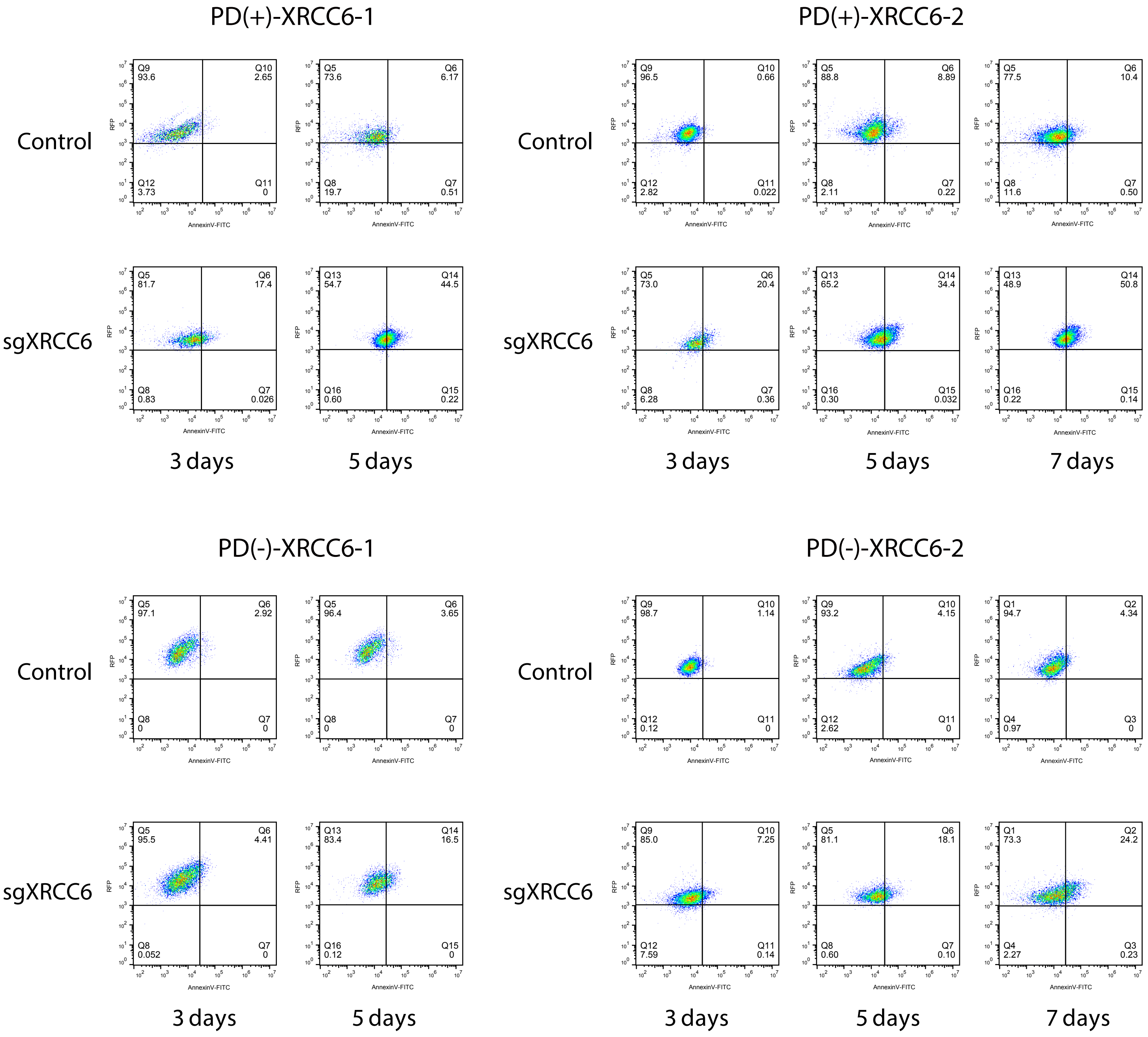

Figure S8B

BrdU

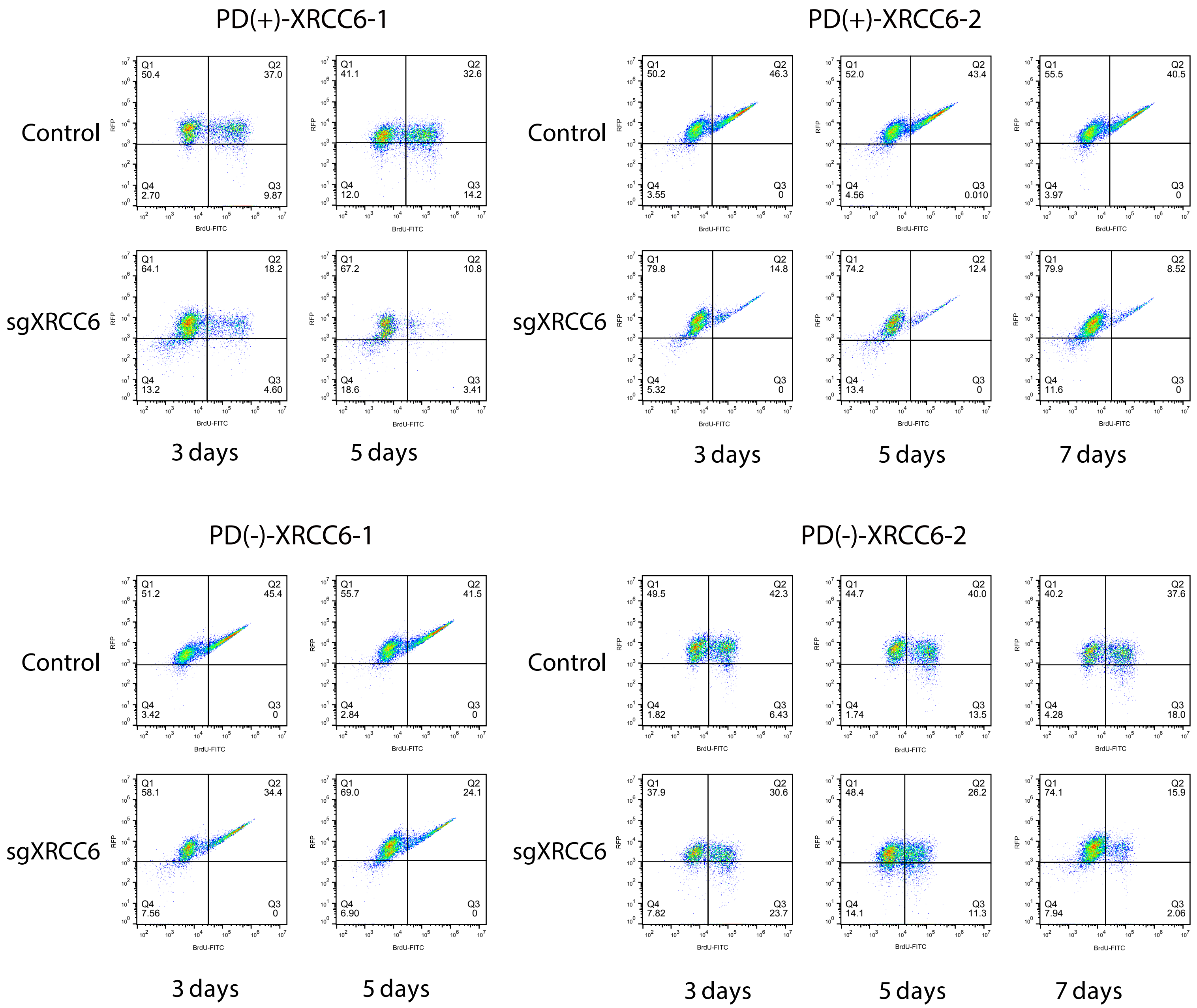

Figure S8. Additional Annexin V and BrdU data for XRCC6.
Scatter plots for (A) Annexin V and (B) BrdU staining in PD(+/–)XRCC6-1 on day 3 and 5 after XRCC6 inactivation and PD(+/–)XRCC6-2 on day 3, 5, and 7 after inactivation. Two sgRNAs were used simultaneously.

Figure S9A

Annexin V

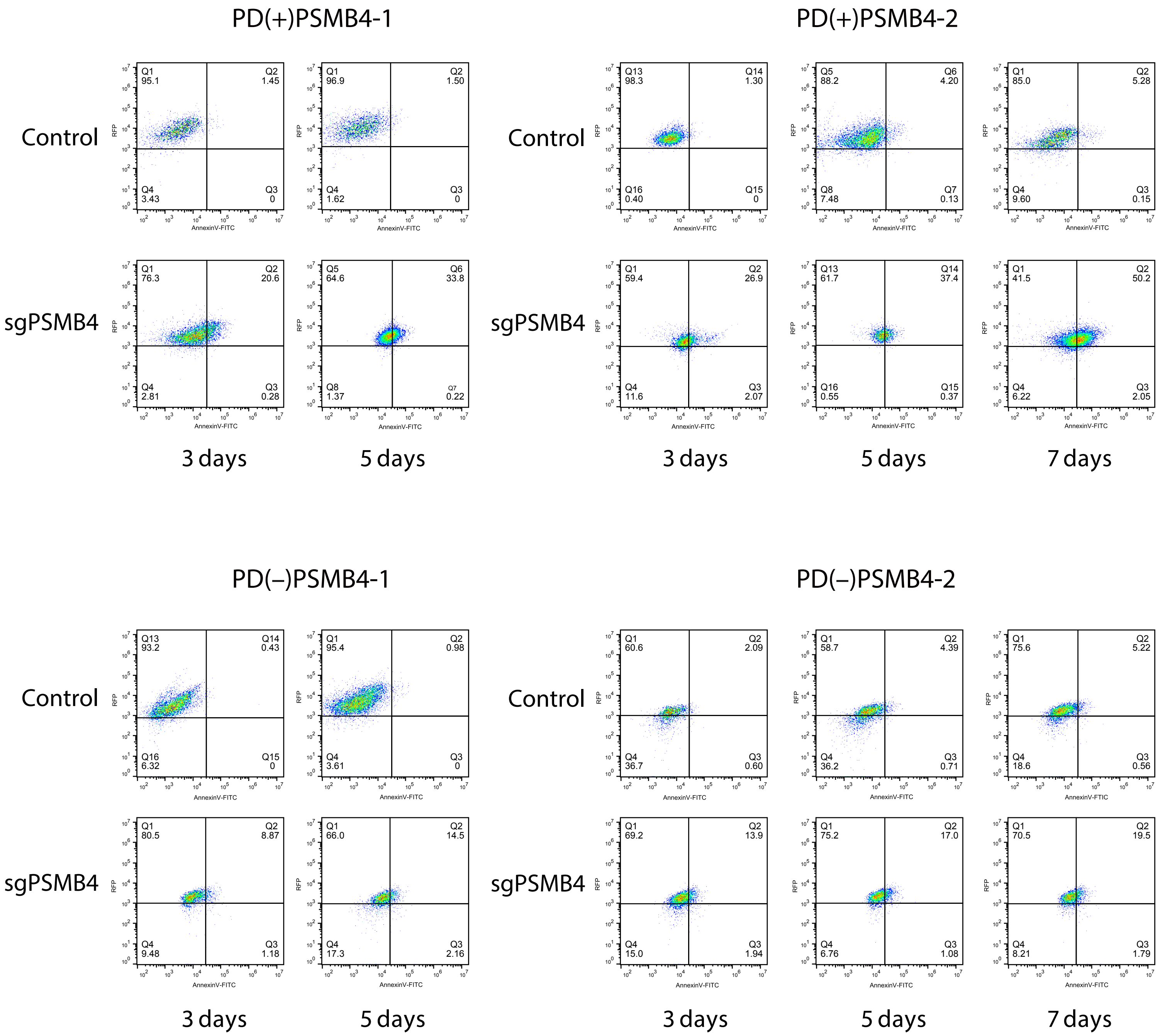

Figure S9B

BrdU

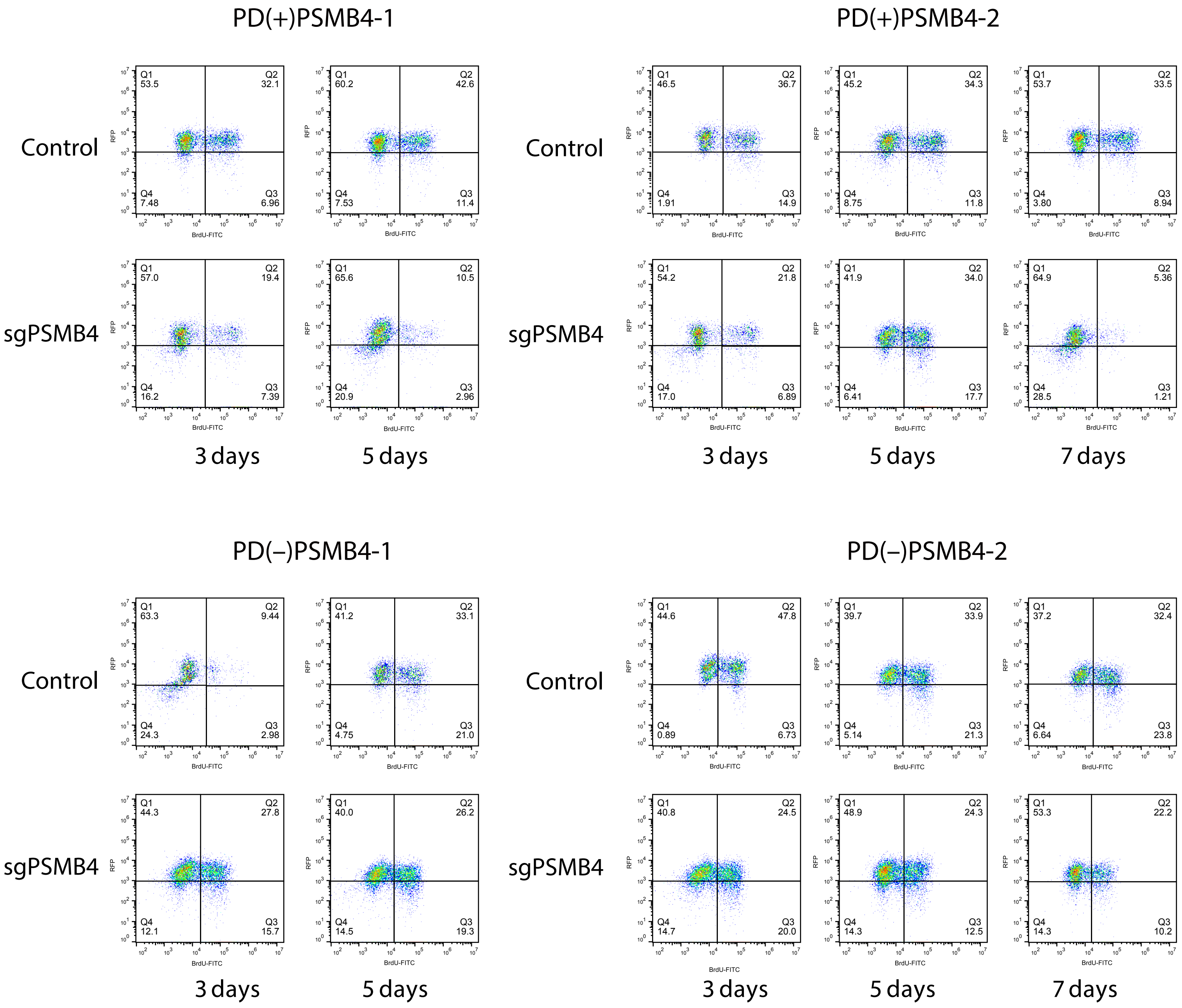

Figure S9. Additional Annexin V and BrdU data for PSMB4.
Scatter plots for (A) Annexin V and (B) BrdU staining in PD(+/–)PSMB4-1 on day 3 and 5 after PSMB4 inactivation and PD(+/–)PSMB4-2 on day 3, 5, and 7 after inactivation. Two sgRNAs were used simultaneously.

Figure S10

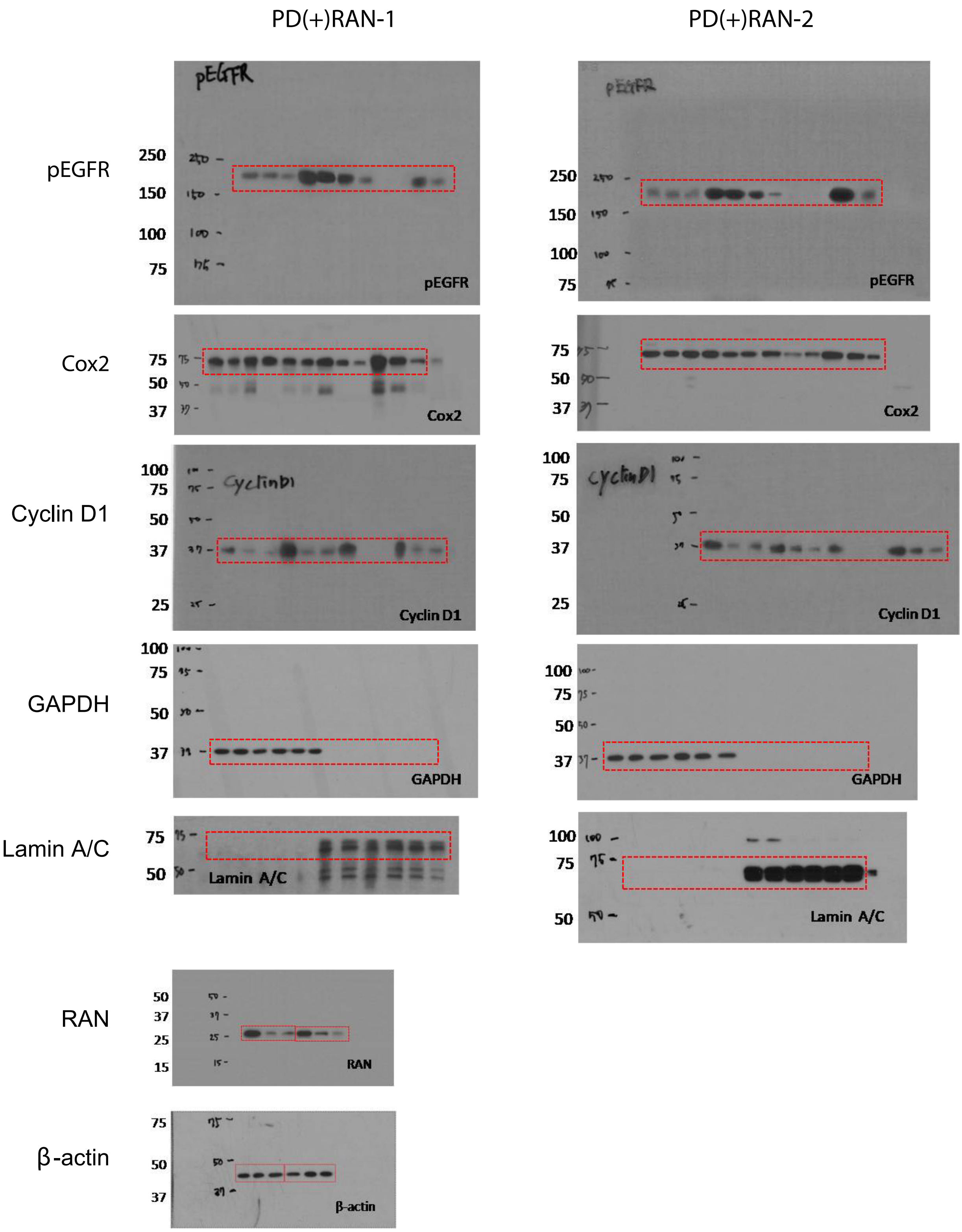

Figure S10. Images of the full uncropped scans for Figure 7A and Figure 7B.

Figure S11

A

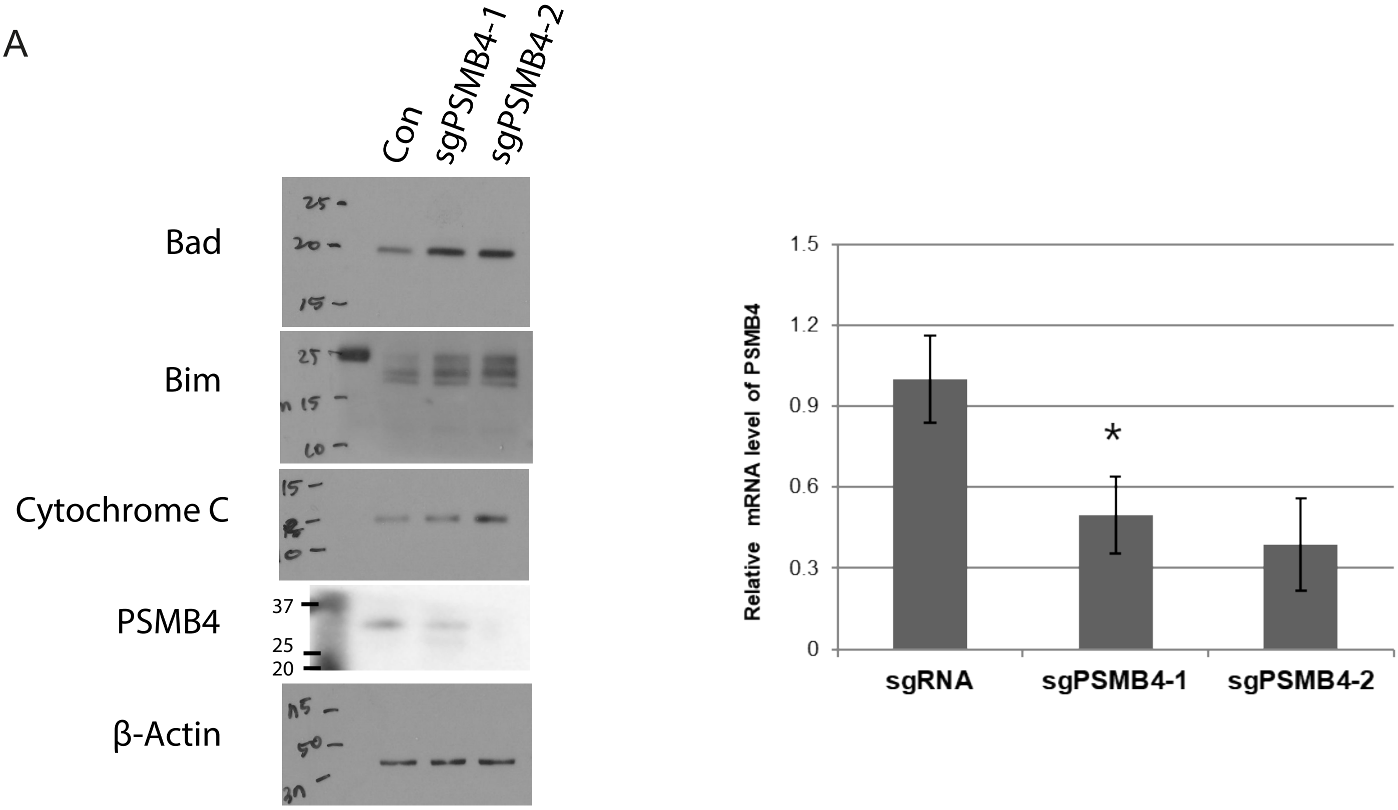

B

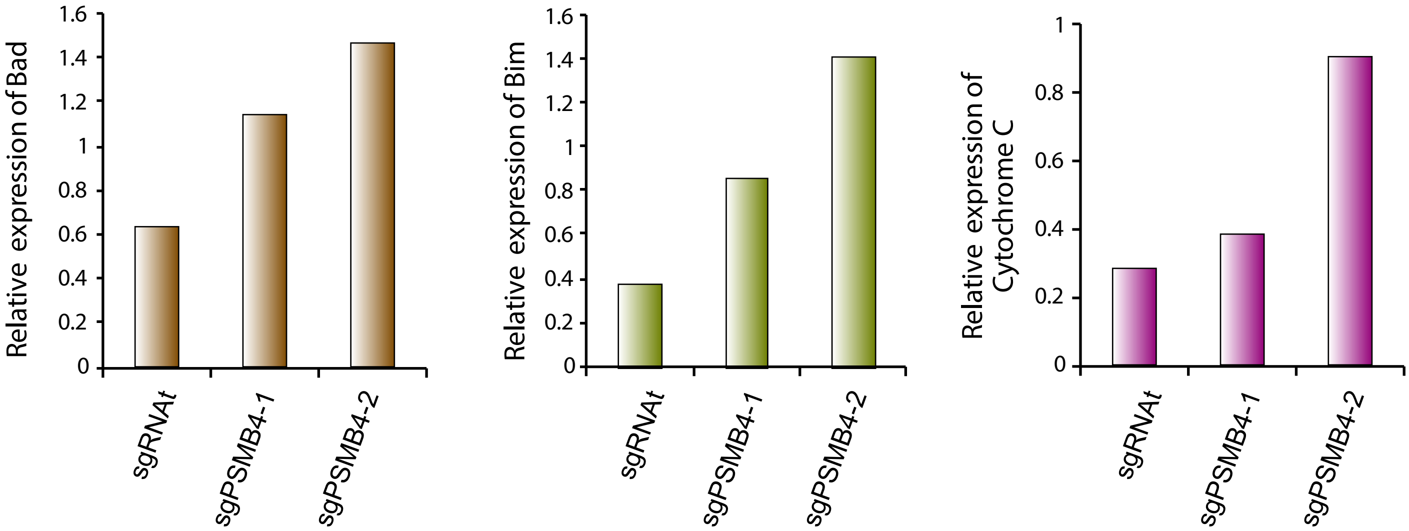

Figure S11. Increases in the level of proapoptotic proteins by PSMB4 knockout.
(A) The western blot results (left) of PD(+)PSMB4-1 targeted with two PSMB4 sgRNAs. Along with reduction in PSMB4 expression (confirmed by real time PCR on the right), the protein level of Bad, Bim, and Cytochrome C was increased. (B) Quantification of the level of the three proapoptotic proteins measured by western blotting.

Figure S12

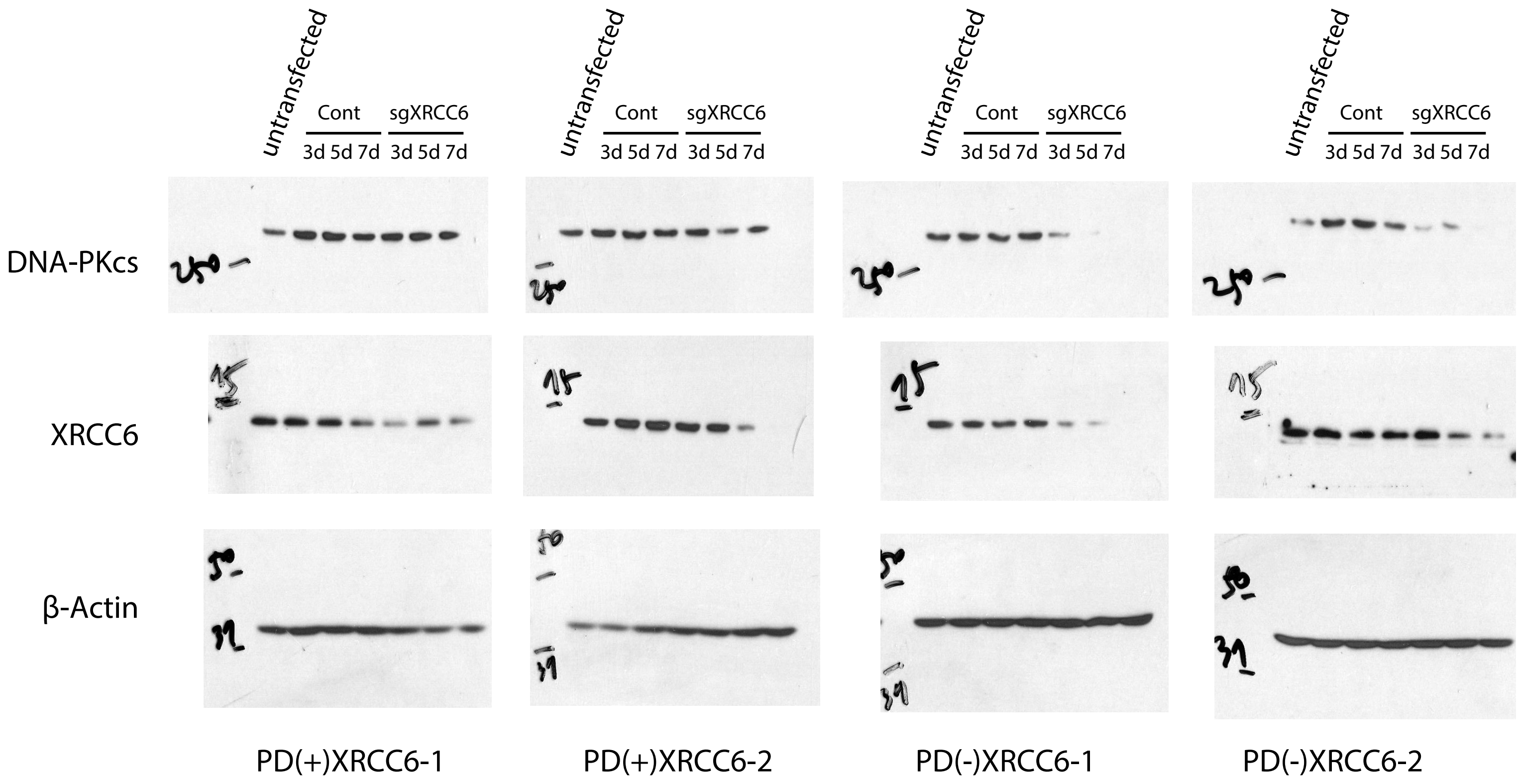

Figure S12. Changes in DNA-PKcs expression in response to XRCC6 knockdown.
Western blot results for DNA-PKcs in PD(+/–)XRCC6-1/2 upon KRCC6 inactivation by sgRNA treatment.

Figure S13

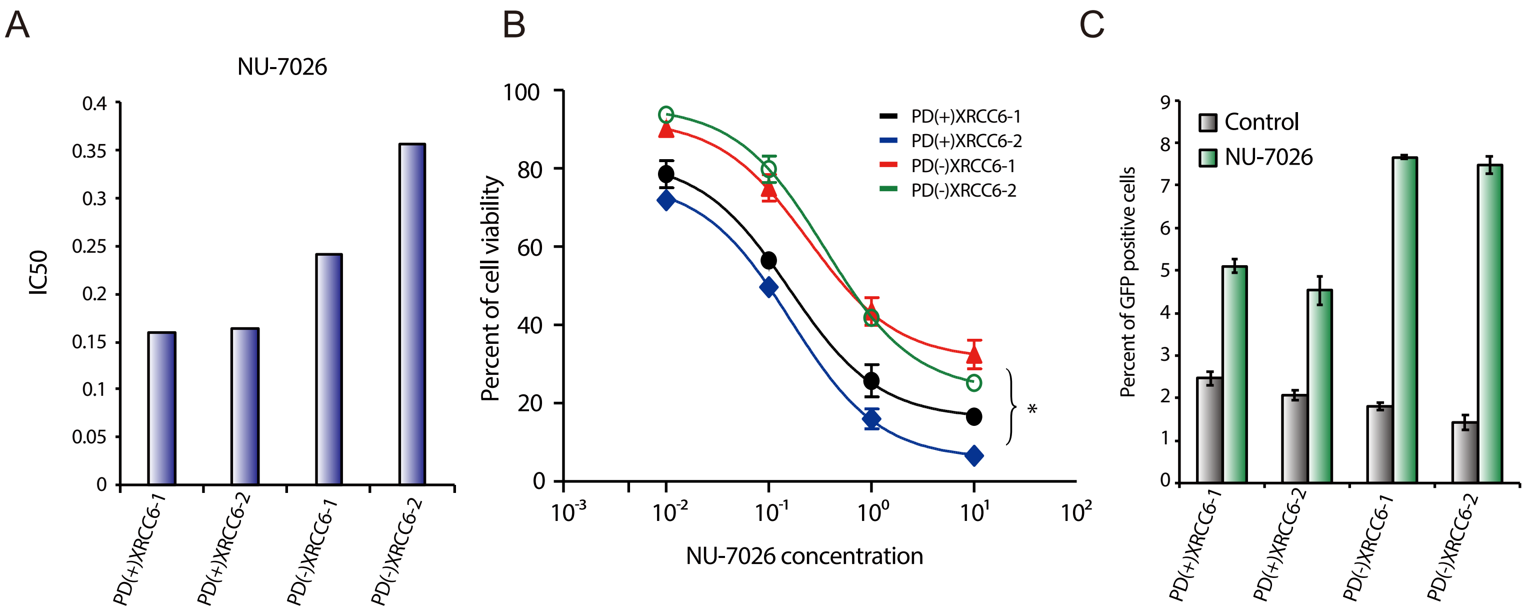

Figure S13. Differential sensitivity to a DNA-PK inhibitor.

(A) IC50 for NU-7026 in PD(+/–)XRCC6-1/2. (B) Cell viability of PD(+/–)XRCC6-1/2 according to different concentrations of NU-7026. (C) The activity of HR repair based on the DR-GFP/I-SceI assay for NU-7026-treated PD(+/–)XRCC6-1/2 cells. Plotted are the percentages of the GFP positive cells.
